# Polymer Brush-Enhanced Extraction and Spreading of Oil from Lubricating Greases

**DOI:** 10.1007/s11249-026-02138-9

**Published:** 2026-04-11

**Authors:** Luciana Buonaiuto, Vincent Siekman, Sander Reuvekamp, Piet M. Lugt, Frieder Mugele

**Affiliations:** 1 Physics of Complex Fluids, MESA+ Institute, University of Twente, PO Box 217, 7500AE Enschede, The Netherlands; 2 Department of Molecules & Materials, MESA+ Institute, University of Twente, PO Box 217, 7500AE Enschede, The Netherlands; 3 Tribology-Based Maintenance, Faculty of Engineering Technology, University of Twente, PO Box 217, 7500AE Enschede, The Netherlands; 4 SKF Research & Technology Development, Meidoornkade 14, 3992AE Houten, The Netherlands

**Keywords:** Grease lubrication, Polymer brushes, Rough surfaces, Wetting, Imbibition

## Abstract

Grease lubrication is among the oldest and most widely employed strategies for reducing friction in tribological contacts. Nevertheless, important microscopic mechanisms that govern the release and transport of lubricants in, say, rolling bearings remain poorly understood. Here, we show how the release of lubricating oil from grease reservoirs and its subsequent spreading toward the rolling contact can be controlled by different treatments of the underlying surface. We demonstrate that smooth, flat oxide surfaces, where oil films are stabilized exclusively by molecular wetting forces, are unable to extract and spread appreciable amounts of oil. In contrast, functionalizing the surface by topographic roughening and/or by coatings of swelling oleophilic polymer brushes can enhance the oil extraction by over two orders of magnitude. Specifically, polymer brushes with an intrinsic phase transition are shown to enable an adaptive lubrication scheme, where the amount of lubricant provided increases rapidly when the temperature rises beyond the melting temperature of the surface-grafted polymer coating.

**Graphical Abstract:**

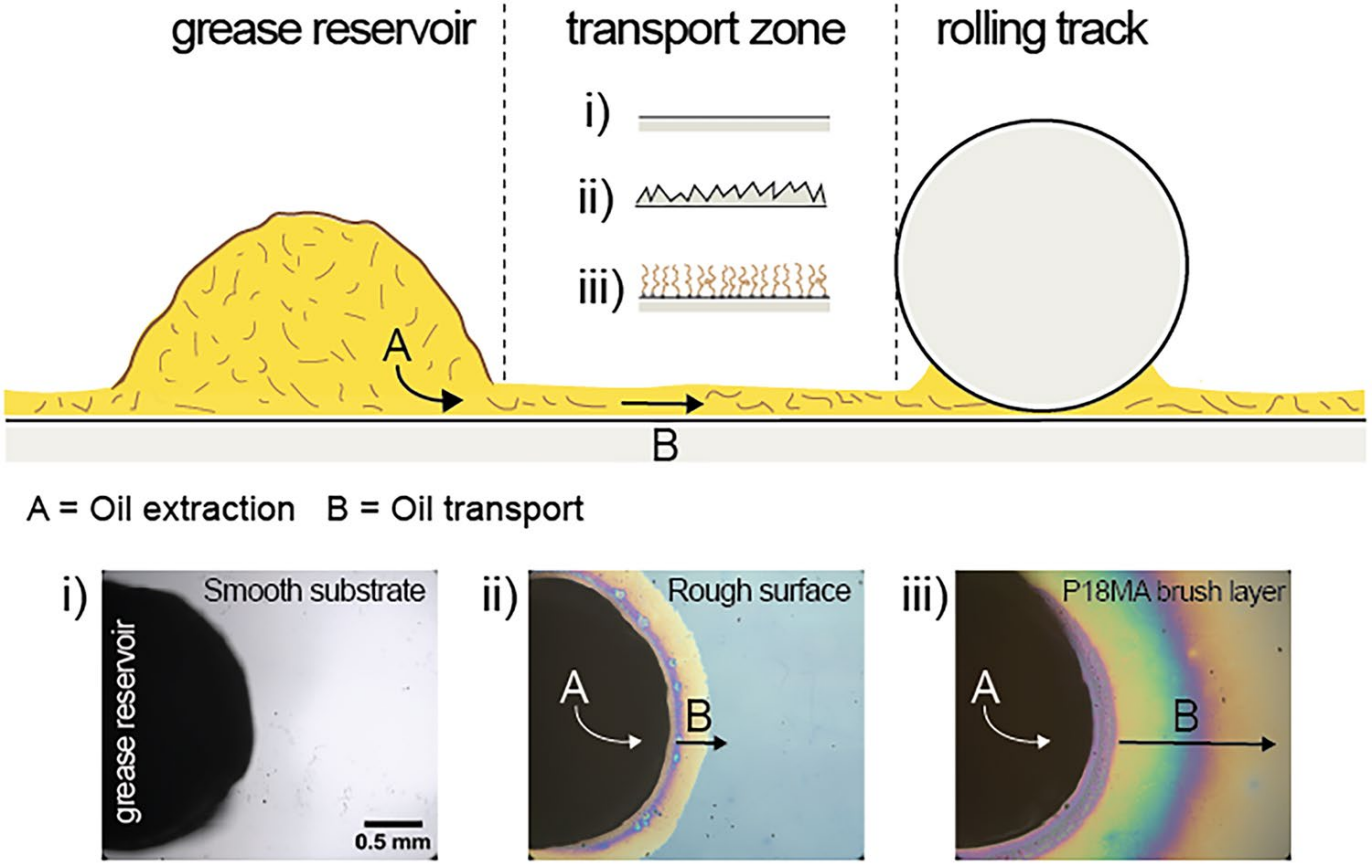

**Supplementary Information:**

The online version contains supplementary material available at 10.1007/s11249-026-02138-9.

## Introduction

Frictional losses represent a major engineering challenge [1–3], accounting for about 30% of global primary energy consumption [4]. In mechanical systems, effective lubrication is essential to reduce friction and wear, and to extend operational lifetime—key factors for a more sustainable future. This is especially critical for rolling bearings which play a central role in many demanding applications, such as wind turbines and electric motors.

**Fig. 1:**
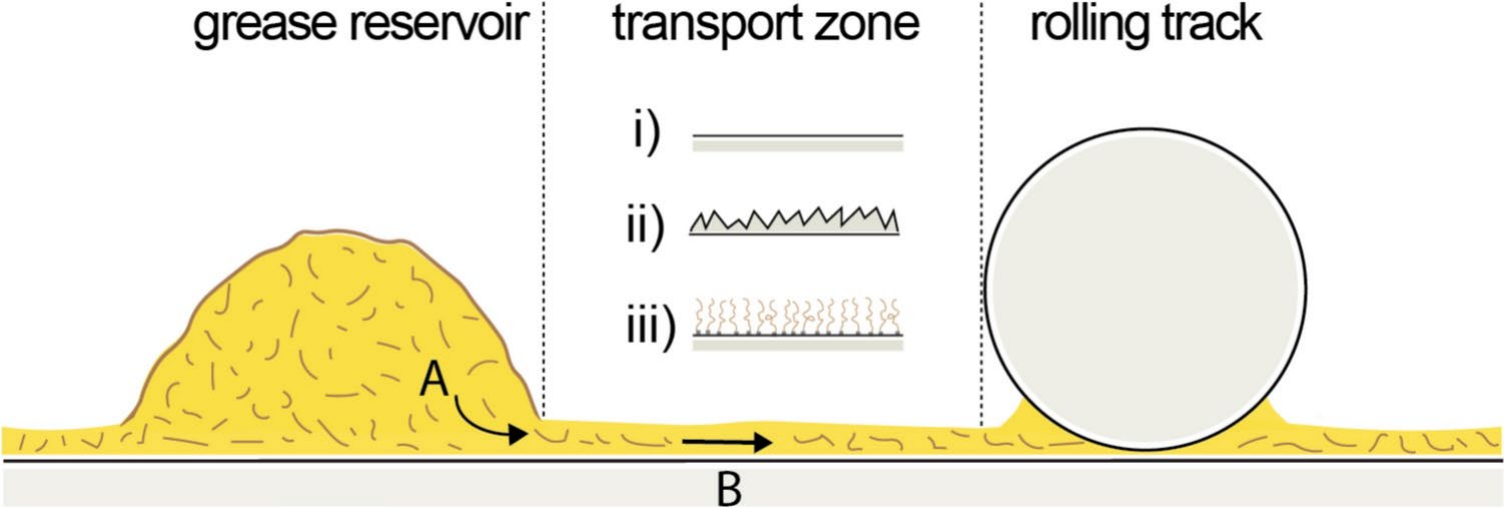
Schematic of a grease-lubricated rolling contact, highlighting three conceptual regions: grease reservoir, transport zone and rolling track and the two main processes: oil extraction (**A**) and oil transport (**B**). For the transport zone, three representative configurations are shown: smooth substrate (i), rough surface (ii), and polymer brush layer (iii)

Grease is the most widely used lubricant in rolling bearings due to its ability to remain in place, operate under sealed conditions, and provide long-term lubrication with little maintenance [5]. Lubricating greases are soft, semi-solid organogels consisting of (often fibrous) networks of thickener that retain the base oil, which ultimately lubricates the tribological contact. During the initial *churning phase*, the grease is distributed throughout the bearing by mechanical motion. Most of the grease is expelled from the *raceway* of the rolling contacts and forms grease reservoirs in adjacent regions such as the bearing shoulders or the cage. Lubrication of the rolling contacts then relies on the release of oil from these reservoirs and on its transport toward the raceway—a process known as *bleeding* [6]. Despite the extensive literature on grease lubrication and bleeding (see, e.g., [5, 7]), little attention has so far been paid to the role of the region between the grease reservoirs and the raceway [8] (see Fig. 1). While the width and geometry of this *transport zone* vary depending on the specific type of bearing, sustained lubrication and good bearing performance can only be achieved if efficient oil transport from the reservoir to the raceway can be guaranteed at all times [5, 9]. As illustrated in Fig. 1, the function of the solid surface in this process is twofold: (A) it extracts oil from the grease and (B) it mediates the transport of the oil from the grease reservoir to the raceway. Function A requires the surface to exhibit a stronger affinity for the lubricating oil than the grease matrix; function B requires that the extracted oil forms a sufficiently thick film on the surface, with a permeability low enough to facilitate the necessary flux.

In fact, the dynamics of oil extraction and subsequent spreading from small patches of grease have been widely studied using porous blotting paper as a substrate (see e.g. [10–12]). Such a process is even used as an industry standard to assess the aging and degradation of lubricating greases. From these studies, it is known that the organogel matrix has an affinity for the base oil that can be expressed as a (negative) retention pressure, typically of the order of a few kPa, which needs to be overcome to extract any oil. Balancing this negative pressure with a typical disjoining pressure arising from molecular interaction forces suggests that the equilibrium thickness of an oil film on a flat surface next to a grease patch should not exceed a few nanometers, even if the oil completely wets the substrate [13]. As a consequence, efficient oil extraction requires either topographic roughness or a specific surface coating that stabilizes much thicker oil films by some form of an ‘effective interaction’ with a much longer range than a conventional disjoining pressure. In the former case, capillary forces drive the liquid into the surface texture, leading to partial or complete wicking of the troughs on the surface [14–18], depending on the balance between surface energy, geometry and the retention pressure in the grease.

To implement the second approach, we explore here the use of polymer brushes, consisting of polymer molecules that are covalently grafted to the surface on one end [19–21]. Unlike rigid topographic patterns, polymer brushes fluctuate under the influence of thermal motion. They can absorb good solvents and swell up to several times their initial thickness. This results in osmotically stabilized layers that consist predominantly of highly mobile solvent molecules. One consequence of this unique behavior is ultra-low friction coefficients that play an important role in (water-based) bio-lubrication [22–24] as well as oil-based lubrication [25–28]. Another consequence of this largely entropically controlled behavior is a pronounced responsiveness of polymer brushes to external stimuli such as temperature and solvent composition. For the present work, we aim to exploit the gain in free energy upon swelling initially dry brushes to extract oil from the grease and the high mobility in the swollen brush facilitates the desired lateral spreading. In previous work, it was demonstrated that oleophilic amorphous polymer brushes of poly-(dodecyl methacrylate) (P12MA) swell by approximately a factor of four and display near-complete wetting upon exposure to *n*-alkanes [29]. Conversely, semicrystalline brushes of poly-(octadecyl methacrylate) (P18MA) act as dry, frozen sponges at room temperature but can be activated to swell and transport oil over hundreds of micrometers once heated above their melting point [30].

In this letter, we demonstrate three qualitative points. First, we confirm that clean and smooth surfaces are indeed unable to extract appreciable amounts of oil from a grease reservoir and are therefore not suitable to support the oil flux required to lubricate a bearing. Second, we show that efficient extraction of oil from a grease reservoir and subsequent spreading can be achieved through suitable surface functionalization. To this end, we fabricate ‘model transport zones’ by coating flat silicon wafers with oleophilic polymer brushes of variable composition and topographic roughness. Finally, we demonstrate that polymer brushes with an intrinsic phase transition allow the design of surfaces that switch from a non-extracting and non-spreading state to an efficient oil-extracting and -spreading state upon exceeding the phase transition temperature.

## Materials and Methods

### Materials

Native oxide silicon wafers (100 ± 0.5 mm diameter, 525 ± 25 μm thickness, boron-doped, orientation, 5–10 Ωcm, OKMETIC) were cut into 1 × 1 cm pieces and used as a substrate for polymer brush growth. The polymerization was performed in 5 mL (40 × 20 mm, Fisherbrand) snap cap glass vials. (3-aminopropyl) triethoxysilane (APTES, 99%, Sigma-Aldrich), triethylamine (TEA, > 99.5%, Sigma-Aldrich), *α*-bromoisobutyryl bromide (BiBB, > 98.0% TCI chemicals), L-ascorbic acid (AA, > 99%, Sigma-Aldrich), *N*,*N*,*N*′,*N*″,*N*″-pentamethyldiethyleentriamine (PMDETA, 99%, Sigma-Aldrich), CuCl2 (97%, Sigma-Aldrich), dodecyl methacrylate (12MA, 96%), octadecyl methacrylate (18MA, 97%, TCI chemicals), ethanol (absolute ≥ 99%, Fisher), toluene (HPLC grade, VWR chemicals), *N*,*N*-dimethylformamide (DMF, 99.8%, Thermo Scientific), Li/M grease (lithium thickener, mineral base oil with base oil viscosity 100cSt at 40 °C; see Zhang et al. [31] for detailed physicochemical properties), *n*-hexadecane (99%, Sigma-Aldrich) were purchased and used without further purification.

### Surface Anchor and Radical Initiator Functionalization

Silicon substrates were sonicated in ethanol for 1 min, rinsed with deionized water and ethanol, and subjected to ozone plasma treatment for 15 min. The activated wafer pieces were immediately placed into a desiccator around a petri-dish containing (3-aminopropyl) triethoxysilane (0.1 mL, 0.43 mmol, APTES). The desiccator was evacuated for 15 min and subsequently closed, allowing for overnight vapor deposition. The amine-terminated substrates were rinsed twice with ethanol and deionized water and blown dry under a nitrogen stream. Along with a stirring bar, the samples were placed face-up into a reaction vessel. In an Erlenmeyer flask, cooled toluene (100 mL) and triethylamine (1.12 mL, 8.05 mmol, TEA) were combined. While stirring vigorously, α-bromoisobutyryl bromide (1 mL, 8.09 mmol, BiBB) was added dropwise. This resulting solution was transferred to the reaction vessel containing the substrates and stirred for 3 h at room temperature. Afterward the substrates were rinsed twice with toluene, ethanol, and deionized water, and dried under a nitrogen stream.

### Brush Polymerization

Polymer brushes of poly(dodecyl methacrylate) (P12MA) and poly(octadecyl methacrylate) (P18MA) were synthesized via Activators Regenerated by Electron Transfer-Atom Transfer Radical Polymerization (ARGET-ATRP) in individual 5 mL snap cap glass vials filled with 7 mL of solution. Although the target molar ratios were similar, the polymerization procedure differed slightly for each brush type:

For the P12MA brushes, two stock solutions are prepared: (1) l-ascorbic acid (43 mg, 244 μmol, AA) in ethanol (10 mL); and (2) CuCl_2_ (28 mg, 210 μmol) in ethanol (10 mL) sonicated to dissolve the CuCl_2_, with *N*,*N*,*N*′,*N*″,*N*″-pentamethyldi-ethyleentriamine (0.1 mL, 21.5 μmol, PMDETA). Into each vial, 4.8 mL of the AA solution (1) was injected, followed by 0.45 of the catalyst solution (2), followed by 1.75 mL dodecyl methacrylate monomer (10.4 mmol, 12MA). The molar ratio of AA/CuCl2/PMDETA/12MA in the reaction mixture was 12.4:1:2.3:597.

For the P18MA brushes, three stock solutions were prepared: (1) AA (74 mg, 420 μmol) in DMF (10 mL); (2) CuCl_2_ (28 mg, 210 μmol) in DMF (10 mL) sonicated to dissolve the CuCl_2_, with PMDETA (0.1 mL, 21.5 μmol); and (3) octadecyl methacrylate (11.9 g, 35.1 mmol, 18MA) in DMF (10 mL) dissolved and kept at 40 °C. Into each vial, 2.7 mL of the AA solution (1) was injected, followed by 0.45 mL of the catalyst solution (2), followed by 3.75 mL of the DMF/18MA (3) solution. The molar ratio of AA:CuCl_2_:PMDETA:18MA in the reaction mixture was 12.0:1:2.3:590.

A functionalized wafer was placed face-up into the vial, which was sealed and left to react for the desired time at room temperature for P12MA and immersed in a 40 °C oil bath for P18MA. Afterward, the polymer brushes were rinsed twice with toluene, ethanol, and deionized water, and dried under a nitrogen stream.

### Rough Surface Preparation

The rough surface was obtained by treating a previously synthesized P18MA brush layer with hexadecane. The sample was immersed in a bath of hexadecane and heated to 40 °C for approximately 10 min. After removal from the bath, excess oil was allowed to evaporate under ambient conditions. This preparation process leads to spontaneous roughening of the sample surface, as shown below. We attribute this phenomenon to a combination of phase separation and crystallization, as we will analyze elsewhere in detail.

### Atomic Force Microscopy

Atomic Force Microscopy (AFM) in tapping mode was employed to characterize the dry thickness and topography of the selected substrates (smooth substrate, rough surface, P12MA brush layer, P18MA brush layer).

AFM meniscus force measurements were recorded in force volume mode with a constant approach and retraction rate of 1 Hz and applied threshold forces of 0.2 nN. The experiments were conducted over a 5 × 5 μm scanning area, collecting 3600 force–distance curves.

All measurements were conducted under ambient conditions using a Bruker Icon system (Santa Barbara, CA, USA) equipped with silicon probes (tip radius *r* < 8 nm, spring constant *k* ≈ 0.6 N m^−1^, HQ: NSC36/Cr-Au BS, MikroMasch).

### Grease Patch Deposition, Oil Extraction, and Spreading

Lithium-based grease (Li/M grease) patches with an approximate diameter of 2.3 mm and a volume of 0.01 mL were manually deposited onto the substrates.

For room temperature experiments, four substrate types were tested: bare silicon oxide surface, rough surface, P12MA brush layer, and P18MA brush layer. The samples were stored under ambient conditions in closed plastic Petri dishes and imaged daily over a period of 6 weeks. For elevated temperature experiments (40 °C), two independent samples—P12MA and P18MA brush layers—were used. The samples were placed on a custom-built heating stage, maintained at 40 °C in open air and imaged daily over a period of 6 weeks.

Optical images were acquired using an upright Zeiss Axioskop El Einsatz microscope (Germany), equipped with a 5× objective (working distance: 13.6 mm) and a Basler acA5472-17uc color camera.

In addition, the quasi-static contact angle (measured several seconds after droplet deposition) of mineral oil, isolated from the lithium-based grease by centrifugation, was measured on the bare silicon oxide substrate and on the P12MA and P18MA brush layers. The measured contact angles were 5°, 22°, and 27°, respectively.

A minimum of three independent samples were tested under each condition to ensure reproducibility. The data and images presented in this study refer to representative experiments selected for clarity and consistency.

### Brush Height Profile

Swelling profiles were obtained using a custom-written MATLAB script to convert the observed color variations into local brush thickness values. The ideal spectral reflectance of an air–brush layer with variable thicknesses on a silicon substrate was simulated and subsequently converted to color coordinates using the transfer matrix method [32, 33]. A chromaticity matching function was then applied to simulate the observed brush color for each thickness, thereby generating a standard color map. The actual colors of the swollen brush layers were matched to the standard color map to determine the thickness for each pixel. Color matching was performed using the ΔE CIE76 method in the CIELAB color space, with the known dry brush thickness obtained from ellipsometry serving as a reference.

## Results and Discussion

### Design and Characterization of Transport Layers

A set of model surfaces with varied topography and chemistry was prepared, including a bare and smooth silicon substrate, a roughened polymer brush surface expected to enhance capillary driven oil flow; and two oleophilic polymer brushes, namely amorphous poly(dodecyl methacrylate) (P12MA) and semicrystalline poly(octadecyl methacrylate) (P18MA).

**Fig. 2:**
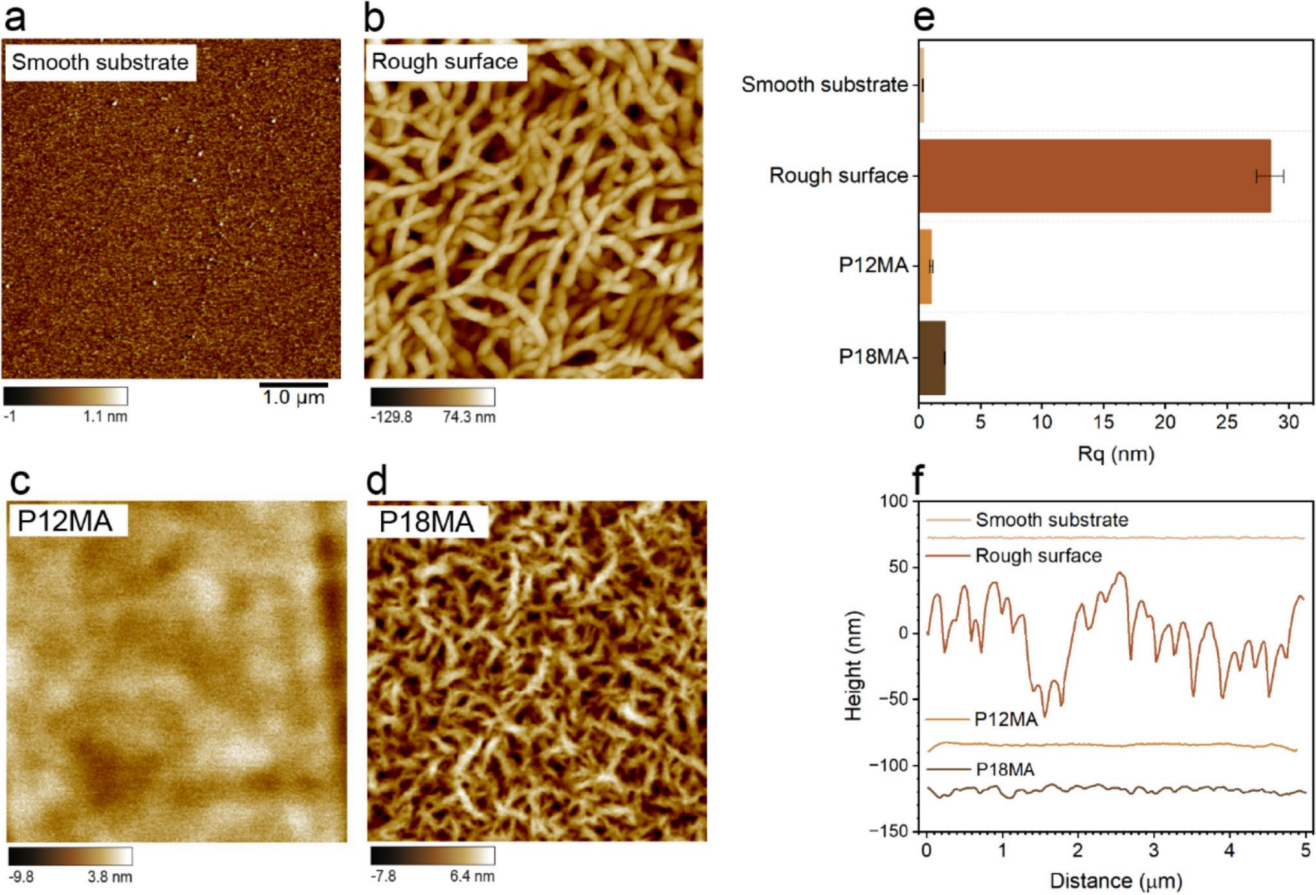
AFM characterization of the transport zone configurations. AFM height images of **a** smooth substrate, **b** rough surface, **c** amorphous P12MA brush layer, and **d** semicrystalline P18MA brush layer. (Note the different color scales). The scale bar is identical for images **a**–**d**. **e** Root mean square roughness (Rq) extracted from AFM topographies shown in panels **a**–**d**. Values represent mean ± standard deviation from four regions of interest. The smooth substrate and the amorphous P12MA brush layer exhibit low surface roughness, while the P18MA brush shows slightly increased roughness due to its semicrystalline morphology. The intentionally roughened surface displays significantly higher surface roughness. **f** AFM cross-sectional profiles (from **a**–**d**), showing surface height variation along the *x*-direction for the four transport zone configurations. The rough surface exhibits large-amplitude features, whereas the bare and brush-coated surfaces show minimal topographical modulation

Atomic force microscopy (Fig. 2a–d) was employed to characterize the topography of the prepared model substrates. The silicon substrate (Fig. 2a) is smooth and largely featureless, while the roughened surface (Fig. 2b) shows pronounced height variations that form potential channels and reservoirs for liquid retention (see Sect. 2 for details about the preparation procedure). This morphology resembles collapsed layers of grease thickener fibers observed in actual bearings, with comparable diameters but distinct architecture. While grease fibers form a three-dimensional network, our engineered roughness is a two-dimensional topographic pattern. P12MA brushes (Fig. 2c) form a very uniform, amorphous layer, expected to exhibit strong affinity for oil [29, 34]. In contrast, P18MA brushes (Fig. 2d) display periodic nanoscale domains characteristic of semicrystalline ordering—features likely to introduce thermally responsive transport pathways [30].

Quantitative analysis of the AFM images (Fig. 2e) reinforces these distinctions. For each substrate, the root-mean square roughness (Rq) was calculated as the average over four distinct 2.5 × 2.5 µm regions selected from a single 5 × 5 µm height image. Rq increases from ~ 0.2 nm for the smooth substrate to ~ 25 nm for the rough surface, with intermediate values for the polymer brushes. Notably, P18MA is slightly rougher than P12MA, consistent with its semicrystalline morphology [35–37].

To further illustrate these differences, cross-sectional profiles were generated by tracing 5 µm-long horizontal lines across a representative region of the height images (Fig. 2f). The resulting profiles highlight the contrast among the four substrates, reinforcing the observed trends in roughness. By establishing this controlled library of topographies and chemistries, we can directly investigate how nanoscale surface features modulate bleeding-driven oil transport.

### Oil Extraction and Spreading Behavior on Functionalized Surfaces

To explore the extraction of oil from grease and its subsequent spreading on the substrate, we deposited patches of grease (diameter: 2.3 mm; height: 2.4 mm) onto the four different substrates. Samples were stored under ambient conditions and optical top-view images were recorded daily over 6 weeks. For the brush-coated substrates, the initial dry thickness in the dry and collapsed state ranges from 100 to 150 nm; while the rough surface exhibits an initial thickness of ~ 200 nm (Fig. S1).

**Fig. 3:**
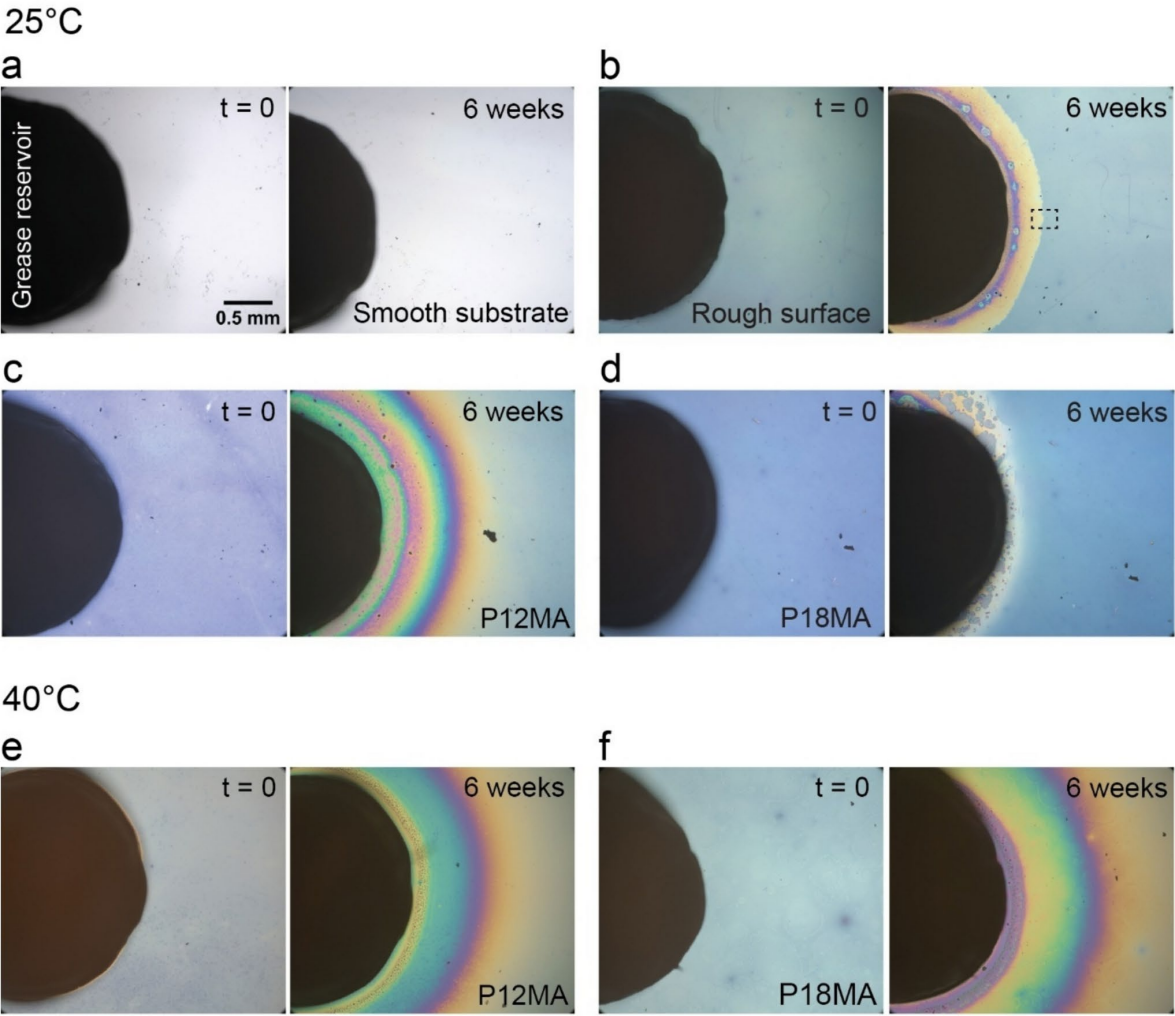
Time- and temperature-dependent extraction and spreading of oil on functionalized surfaces. Optical top-view images of cylindrical Li/M grease patches (black) deposited onto four different transport zone configurations: **a** smooth substrate, **b** rough surface, **c** amorphous P12MA brush layer, and **d** semicrystalline P18MA brush layer. Left: immediately after grease deposition; right: after 6 weeks of storage at room temperature. Cylindrical Li/M grease patches (black) deposited onto P12MA and P18MA brush layers heated at 40 °C (above the melting transition of P18MA): **e** P12MA and **f** P18MA. Left: immediately after deposition; right: after 6 weeks at 40 °C. The scale bar is identical for images **a**–**f**. (See Figs. S2–S7, Supporting information for additional data)

On the smooth substrate (Fig. 3a), grease patches remained sharply defined, with no noticeable lateral spreading of oil. This behavior is consistent with a partial wetting regime, where the oil may form an ultrathin precursor film—on the order of at most a few nanometers—stabilized by disjoining pressure and molecular interactions. However, such a film can sustain only minimal oil flux over extended periods [38, 39]. As a result, oil extraction and film formation on a smooth substrate remain negligible, severely limiting lubricant supply.

In contrast, both the rough surface (Fig. 3b) and the P12MA brush layer (Fig. 3c) develop a colorful halo surrounding the grease patch quickly after the deposition. For the rough surface, the homogeneous yellowish color of the rim indicates that the degree of oil impregnation is very homogeneous—except for a narrow region close to the edge of the grease patch. This halo reflects spontaneous uptake and lateral migration of oil along the surface. Both mechanisms will be described in greater detail in the following section. At elevated temperatures, halo formation on P12MA (Fig. 3e) is further enhanced. We attribute this to an increase in oil diffusivity at elevated temperature, which facilitates imbibition along the oleophilic brush surface.

The semicrystalline P18MA brush exhibits a distinct behavior: at room temperature, grease patches remain largely unchanged after 6 weeks (Fig. 3d), with negligible oil extraction. In contrast, a pronounced halo appears above the melting transition (Fig. 3f), implying significant oil extraction and lateral spreading. As previously demonstrated, P18MA brushes do not respond to the ambient fluid below their melting point. However, once heated above the transition, the brush layer becomes active, enabling fluid sorption and lateral diffusion [30]. This response can be attributed to the inability of solvent molecules to disrupt the crystalline domains of P18MA, likely due to thermodynamically unfavorable conditions for oil absorption below the melting transition.

### Capillary Flow and Brush Swelling Dynamics

**Fig. 4:**
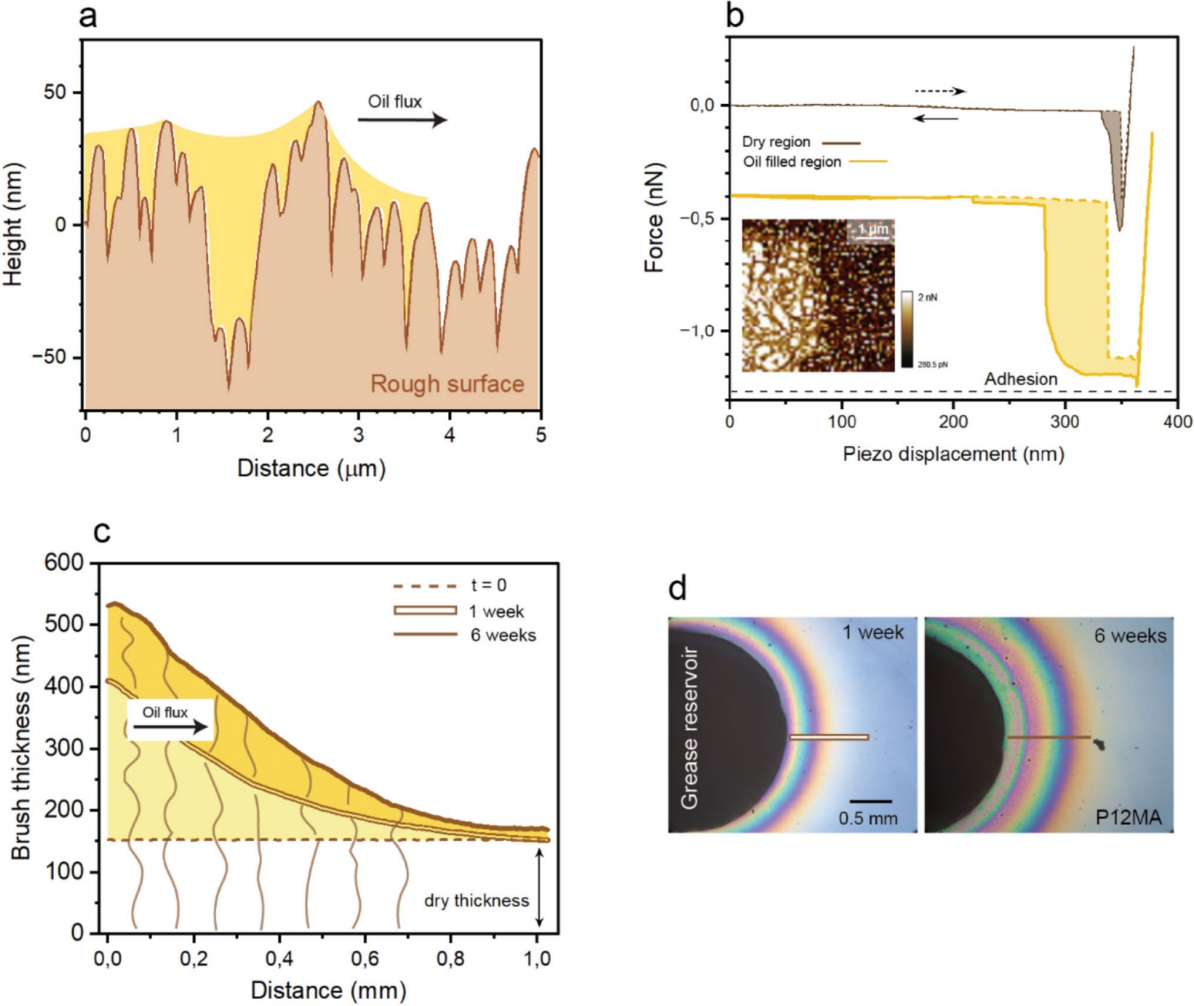
Oil transport mechanism on functionalized surfaces. **a** Surface topography of the rough surface extracted from AFM measurements, with a schematic illustration of oil-filled valleys and grooves (yellow). **b** Representative force–distance curves recorded in force–volume mode on the rough surface within the dry region (top; brown) and the oil-filled region (bottom; yellow), vertically offset for clarity. Dashed curves: approach; solid curves: withdrawal. Inset: adhesion map derived from force–volume data for the area indicated by the dashed square in Fig. 3b; dark: dry region with lower adhesion; brighter: oil-infused region. Lateral variations within both regions correlated with the surface topography (see Fig. S8, Supplementary information for additional data). **c** Evolution of the P12MA brush thickness profile extracted along the double line (left) and the solid line (right) indicated in panel **d**, obtained from white-light interferometry. The dry brush (dashed line), after 1 week (double line), and after 6 weeks (solid line) illustrate the progressive swelling and lateral spreading of oil over time. **d** Optical top-view images of cylindrical Li/M grease patches (black) on the P12MA: left, after 1 week at room temperature; right, after 6 weeks. The scale bar is identical for both images (Color figure online)

Understanding the mechanisms that govern oil transport is key to interpreting the amount of oil extracted from grease on different substrates. On rough substrates, oil transport is strongly influenced by capillarity within the topographical features. When a drop of a wetting liquid is placed on a textured surface, the liquid can penetrate the surface grooves, advancing ahead of the drop contact line. This phenomenon, known as wicking or hemiwicking, arises from capillary-driven imbibition of fluid into the roughness grooves [40, 41]. In our experiments, the appearance of a halo around the grease patch can be attributed to such oil infiltration into the microstructured network, with favorable oil–surface interactions further enhancing transport (Fig. 4a).

To probe this mechanism more directly, we performed AFM meniscus force measurements following the approach of Peppou–Chapman and Neto [42]. To compare oil-filled and dry regions, we scanned an area near the halo edge (dashed square in Fig. 3b). The resulting force–volume data reveal distinct force–distance behaviors depending on the presence of oil in the surface roughness (Fig. 4b).

In dry regions, curves resemble those typical of a hard solid substrate (upper curves in Fig. 4b). During approach, the cantilever remains undeflected until a few nanometers from the surface, when a sudden jump-to-contact occurs. Further indentation leads to a steep, nearly linear upward deflection of the cantilever, reflecting the repulsive response of the hard substrate. During retraction, the cantilever deflects downward, indicating a finite adhesive interaction. At larger separations, the adhesion is overcome, and the cantilever snaps back to its undeflected position, with a pull-off force of approximately 0.2 nN.

In oil-filled regions, the force–distance response differs markedly from that observed on dry areas (lower curves in Fig. 4b). At relatively large tip–surface separations, the cantilever remains undeflected until it encounters the thin oil layer, which induces an early jump-to-contact. As previously reported [42], this behavior is caused by the formation of a capillary meniscus between the AFM tip and the liquid film, which pulls the cantilever downward before direct contact with the solid substrate. As the approach continues, the tip eventually reaches the underlying surface, marked by the transition to positive cantilever deflection. Upon retraction, the presence of the liquid layer leads to stronger adhesive interactions than in dry regions. A capillary bridge forms between the tip and the oil-covered surface and is progressively stretched during withdrawal, resulting in a prolonged attractive regime extending over distances of approximately 100 nm. Detachment occurs only after rupture of this capillary bridge, giving rise to an increased pull-off force characteristic of liquid-mediated adhesion. The contrast between dry and oil-filled regions is further highlighted by the adhesion map shown in the inset of Fig. 4b.

In contrast, polymer brushes absorb oil through a characteristic swelling-driven mechanism. Upon contact with the grease patch, the initially dry P12MA brush rapidly absorbs oil due to its oleophilic character as well as the gain in mixing entropy. Fluid is initially sorbed at the solid–liquid interface and subsequently imbibed into the polymer layer, where swelling is governed by the balance between osmotic driving forces and the elastic resistance of the tethered chains. This balance reflects the interplay between entropic gains, arising from favorable oil–polymer mixing, and entropic penalties associated with chain stretching. Swelling thus represents the point at which the energetic cost of chain stretching compensates the free energy gain from solvent absorption [43]. As the brush swells, lateral diffusion within the layer promotes the redistribution of oil beyond the immediate contact region, giving rise to a colorful halo of partially swollen polymer surrounding the grease patch (Fig. 4d). The behavior of the extracted base oil from the grease closely mirrors the spreading of hexadecane drops previously reported on the same brushes [29], where the liquid rapidly penetrated the dry layer and generated a pronounced interference halo. In the present case, the grease patch acts as a continuous oil reservoir, supplying fluid that is sorbed into the brush and transported laterally within the swollen zone. The progressive increase in oil content is directly visualized by the evolution of interference colors under white-light illumination (Fig. 4d; see Figs. S4, S6–S7, Supporting Information for additional data), which can be quantitatively converted into brush thickness profiles (Fig. 4c; see Sect. 2).

### Quantification of Oil Extraction

Having established the distinct oil transport mechanisms, we can now calculate the total amount of oil extracted from the grease. For the rough surface, the extracted oil fills the valleys and grooves of the microstructured topography; the corresponding oil volume can therefore be estimated from the measured roughness profiles (Fig. 4a), by integrating the liquid-filled cross-sectional area over the entire halo region.

**Fig. 5:**
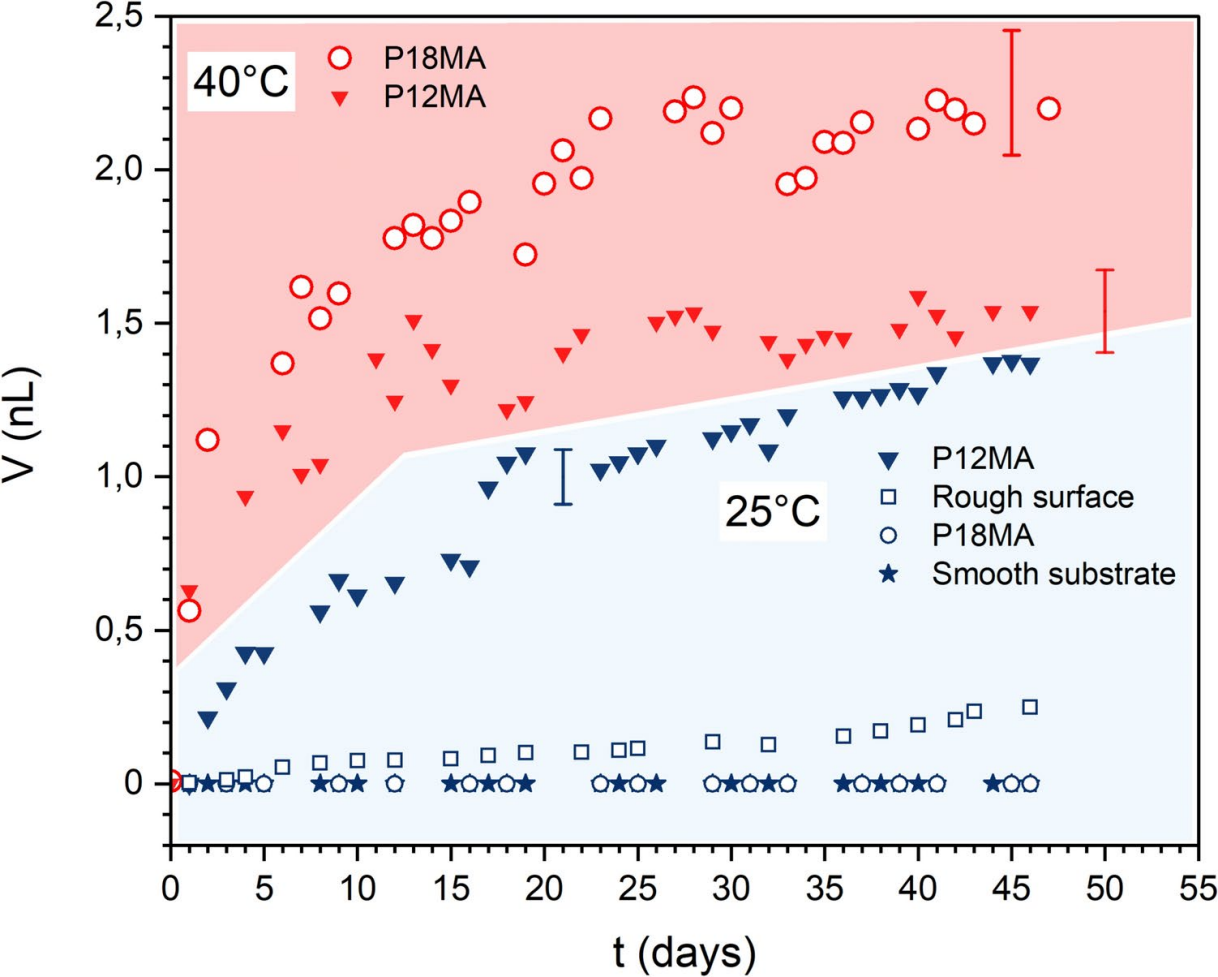
Extracted oil volume over time for the different transport zone configurations. At room temperature (blue region, 6 weeks): bare substrate (stars), rough surface (open squares), amorphous P12MA brush (down triangles), and semicrystalline P18MA brush (open circles). At 40 °C (red region, 6 weeks): oil release is further enhanced on P12MA (down triangles) and activated on P18MA (open circles) (Color figure online)

For polymer brushes, the increase in layer thickness obtained from optical interferometry directly reflects swelling due to oil absorption. Given that polymer and oil have comparable densities, the resulting expansion of the brush volume corresponds to the volume of oil extracted from the grease patch. Integration over the swollen area thus provides the total extracted oil. As shown in the optical microscopy images (Fig. 3), on the bare substrate (stars in Fig. 5) the grease patch remains essentially unchanged over 6 weeks, meaning that the extracted oil volume is negligible and can be considered zero within our experimental resolution. The same holds true for the semicrystalline P18MA brush layer under these conditions (open blue circles). In contrast, the rough surface (open blue squares) shows a clear ability to promote oil extraction, reaching approximately 0.2 nL after 6 weeks. However, the P12MA brush (down triangles) exhibits a strikingly higher extraction capacity—over an order of magnitude greater than the rough surface. These differences become more pronounced at elevated temperature. For the P12MA brush, oil extraction is slightly accelerated due to an increase in oil diffusivity. Nevertheless, after approximately 2 weeks, the amount of oil extracted appears to reach a plateau (red down triangles in Fig. 5). (Additional control experiments suggest that this saturation is probably caused by evaporation of the more volatile components of the base oil. Similar to the earlier studies by Kap et al. [29], further brush swelling is then halted by the balance of continued oil extraction and evaporation from the partly swollen brush layer.)

The most remarkable change, however, is observed for the P18MA brush: below its melting transition (blue open circles), the brush remains impermeable to oil, whereas above the transition (red open circles), the extracted volume increases dramatically from nearly zero to more than 2.2 nL over the same period. This pronounced increase in oil uptake suggests that materials such as P18MA could enable advanced lubrication schemes with an autonomously self-regulating thermoresponsive oil transport (Fig. 5). (Note here that the flexibility of the architecture of polymer brushes allows tuning of the transition temperature over a wide range. For instance, the melting temperature of P22MA is at 55.6 °C [37].)

### Functionalized Surfaces as Active Transport layers

**Fig. 6:**
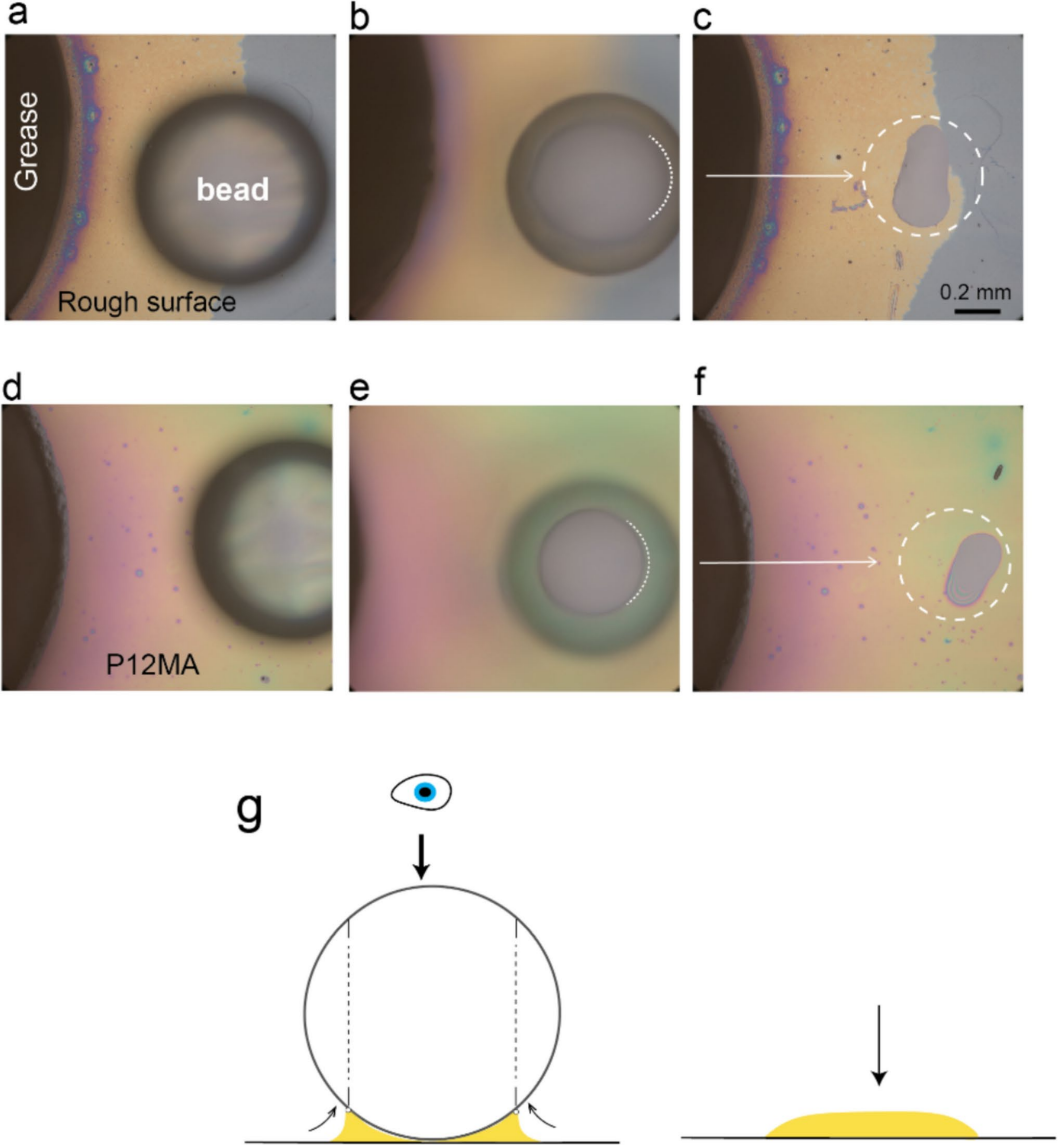
Optical top-view sequences showing a glass bead (*d* ≈ 0.7 mm) resting on (**a**) a rough surface and (**d**) the swollen P12MA brush. The bead–surface contact regions, imaged through the bead, reveal the growth of an oil meniscus (see dotted lines for the edge) at the interface (**b**–**e**). Following bead removal after 10 days in contact (**c**–**f**), a residual oil droplet indicates the formation and subsequent rupture of the capillary bridge. **g** Side-view schematic illustrating the oil meniscus formation at the bead-surface interface (left) and the residual droplet after bead removal (right), as experimentally observed. The scale bar is identical for images (**a**–**f)**

To illustrate that the oil absorbed into the transport layer can also be extracted at another location, we placed a glass bead (*d* ≈ 0.7 mm) on both the rough surface (Fig. 6a) and on the P12MA brush layer (Fig. 6d), approximately 1 mm away from the edge of the grease patch. This sphere can be thought of as mimicking a bearing ball in contact with the brush layer. Upon depositing the bead on the surface, excess oil accumulates around the contact point between the bead and the substrate. The presence of the resulting capillary bridge becomes evident by adjusting the focal plane and imaging through the glass bead (Fig. 6b–e, see Ref. [44] for details on the microscopy method). This observation indicates that the bead can trigger oil extraction via capillary forces from both the underlying liquid-infused rough surface (top row) and the swollen brush layer (bottom row). After bead removal, following 10 days in contact, a thin residual droplet was observed at the contact region on both surfaces (Fig. 6c and f), providing further evidence of a capillary bridge that formed and subsequently ruptured. These results indicate that, despite their structural differences, both the rough surface and the swollen brush layer can extract oil from the grease patch and release it at the bead–surface interface. On the rough surface, oil is first drawn into the surface asperities, where micro-menisci with a higher (negative) curvature generate a lower Laplace pressure than within the grease matrix, enabling oil extraction. The oil then spontaneously infuses the roughness and is transported laterally along the surface. Upon contact with the glass bead, a meniscus with an even higher curvature forms at the bead–surface interface, generating an even stronger capillary suction that pulls oil out of the surface and into the contact region. An analogous mechanism operates for the swollen brush layer. Oil is initially absorbed within the brush due to its higher affinity for the lubricating oil compared to the grease matrix. As the brush swells, oil is laterally redistributed within the layer. When the bead is brought into contact, the capillary forces at the interface exceed the retention capability of the swollen layer, causing oil to be released and to accumulate beneath the bead. Together, these observations demonstrate that functionalized surfaces not only extract oil from the grease reservoir and spread it across the surface but can also release it and thereby lubricate a third body in contact with the surface.

## Conclusion

Our experiments demonstrate that the extraction of oil from a grease reservoir and its subsequent spreading along the surface are controlled by the interaction of the oil with the underlying solid surface. While smooth substrates are unable to extract appreciable amounts of oil, both topographic roughening and coatings of swellable polymer brushes enable oil extraction and lateral transport over millimeters. For the conditions under investigation, the rate of oil extraction and spreading was found to be most pronounced for the swellable polymer brushes, presumably due to the very strong sorption capacity of the initially dry brush. Moreover, both the liquid-infused rough surface as well as the swollen brush layers are capable of subsequently releasing the oil again to a solid sphere mimicking a bearing ball. These observations reveal the sequence of elementary steps that are necessary to ensure sustained lubrication in a grease-lubricated bearing and demonstrate the significance of a functional transport layer that facilitates oil extraction and spreading in a more efficient manner than a bare flat surface. In commercial bearings, this functioning of the transport layer is (most likely) determined by a combination of topographic roughness of the steel surfaces as well as residual thickener fibers from the churning phase that add further to the roughness. Given the randomness of the fiber distribution, it seems plausible that occasional fiber-free regions can lead to poor local lubricant supply along with possible local starvation, excess friction and wear. Potentially, homogeneously grafted polymer brush layers could help to mitigate this problem. Moreover, the tunable transport properties of the P18MA brushes emerging from their thermos-responsiveness suggest a promising generic approach for applications requiring adaptive and self-regulating fluid supply, be it in lubrication or beyond.

## Supplementary Information

Below is the link to the electronic supplementary material.

Supplementary file1 (DOCX 1303 KB)

## Data Availability

All data are available from the corresponding author upon request.
