# Supplementary material for: Polymer Brush-Enhanced Extraction and Spreading of Oil from Lubricating Greases
Source: Tribol Lett. 2026 Apr 11;74(2):44. doi: 10.1007/s11249-026-02138-9 (PMC13070056; doi:10.1007/s11249-026-02138-9)
Supplement: Supplementary file 1 — Supplementary file1 (DOCX 1303 KB) [file 11249_2026_2138_MOESM1_ESM.docx]

**Supporting information**

**Polymer Brush-Enhanced Extraction and Spreading of Oil from Lubricating Greases**

Luciana Buonaiuto*^1^, Vincent Siekman^1^, Sander Reuvekamp^1,2^, Piet M. Lugt^3,4^, Frieder Mugele^1^

1. Physics of Complex Fluids, MESA+ Institute, University of Twente, PO box 217, 7500AE, Enschede, The Netherlands
2. Department of Molecules & Materials, MESA+ Institute, University of Twente, PO box 217, 7500AE, Enschede, The Netherlands
3. Tribology-based Maintenance, Faculty of Engineering Technology, University of Twente, PO Box 217, 7500AE, Enschede, The Netherlands
4. SKF Research & Technology Development, Meidoornkade 14, 3992AE, Houten, The Netherlands

[*l.buonaiuto@utwente.nl](mailto:*l.buonaiuto@utwente.nl)


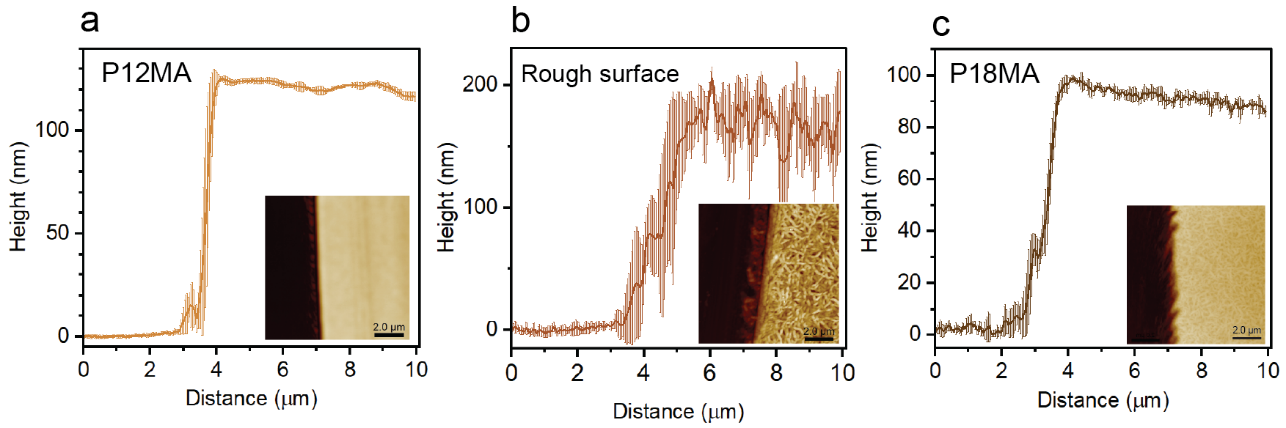


**Fig. S1** Thickness profile of P12MA brushes **(a)**, rough surface **(b)** and P18MA brushes **(c)** measured via AFM in tapping mode. The insets show the corresponding topographic images, highlighting the boundary between the SiO₂ substrate (dark stripe, left) and the brush/rough surface (light stripe, right)

**
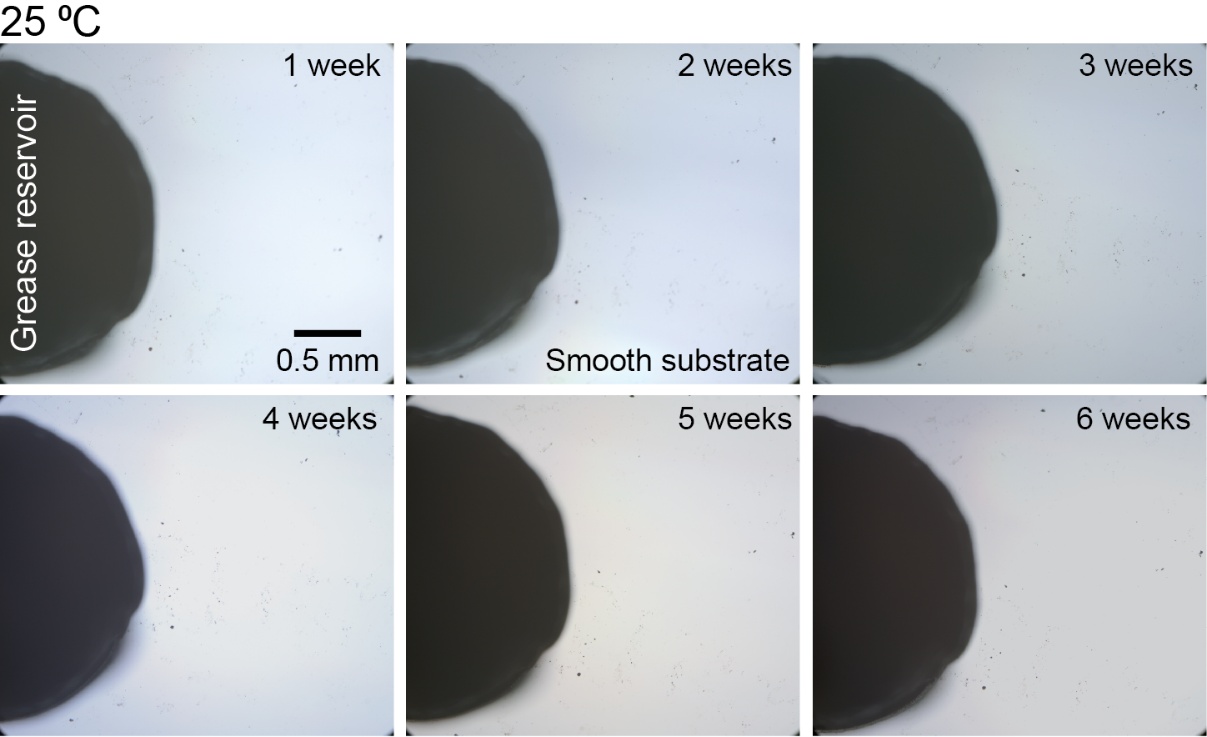
**

**Fig. S2** Optical top-view images of cylindrical Li/M grease patches (black) deposited on the smooth substrate. Samples were stored at room temperature for six weeks; one representative image per week is shown


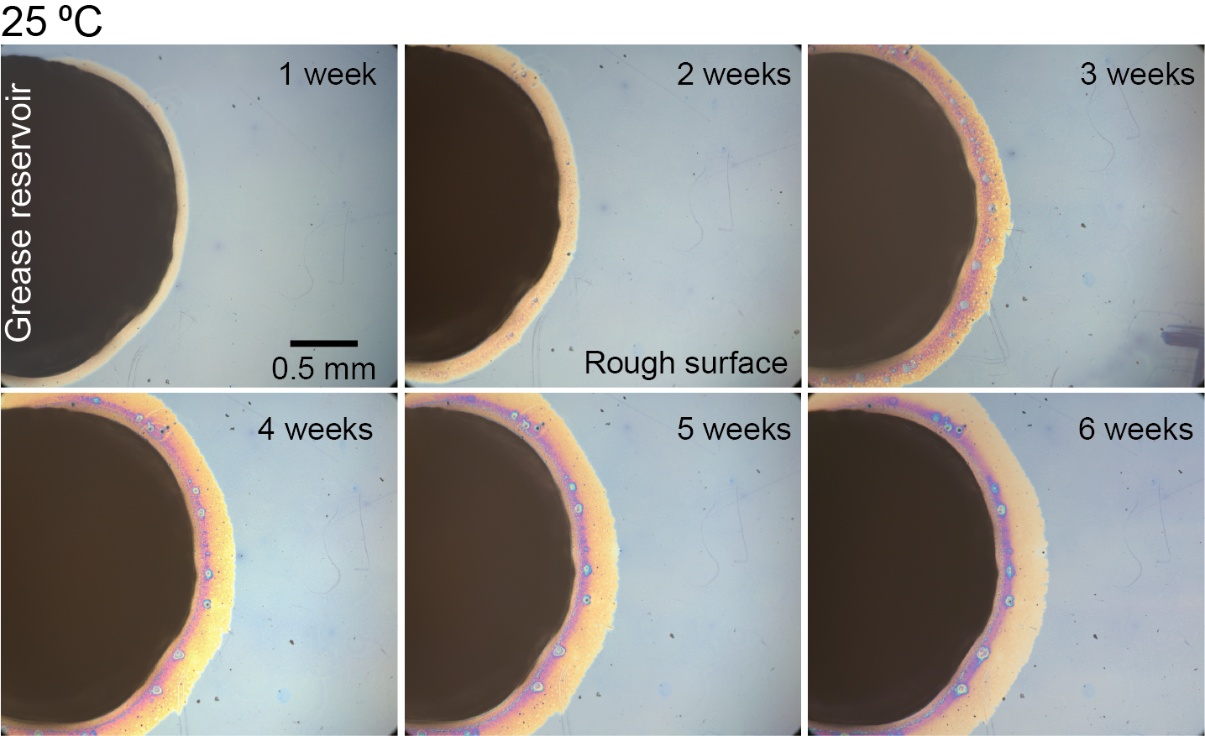


**Fig. S3** Optical top-view images of cylindrical Li/M grease patches (black) deposited on the rough surface. Samples were stored at room temperature for six weeks; one representative image per week is shown


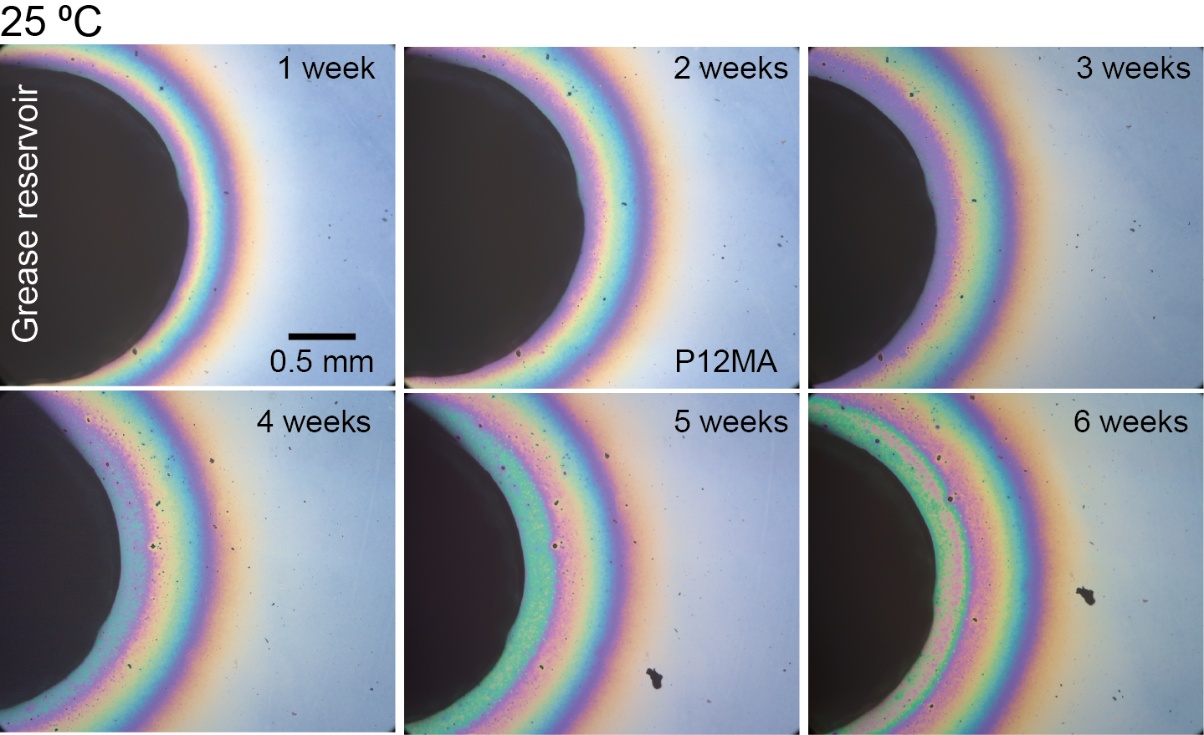


**Fig. S4** Optical top-view images of cylindrical Li/M grease patches (black) deposited on the P12MA brush layer. Samples were stored at room temperature for six weeks; one representative image per week is shown


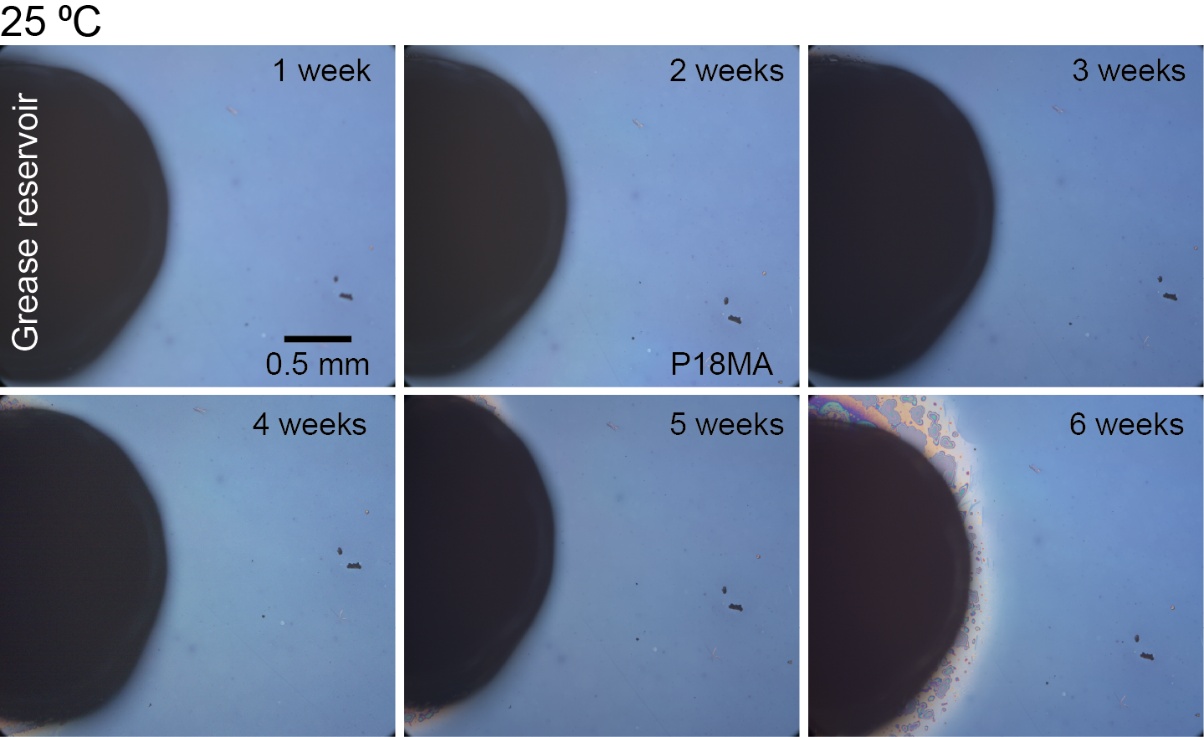


**Fig. S5** Optical top-view images of cylindrical Li/M grease patches (black) deposited on the P18MA brush layer. Samples were stored at room temperature for six weeks; one representative image per week is shown


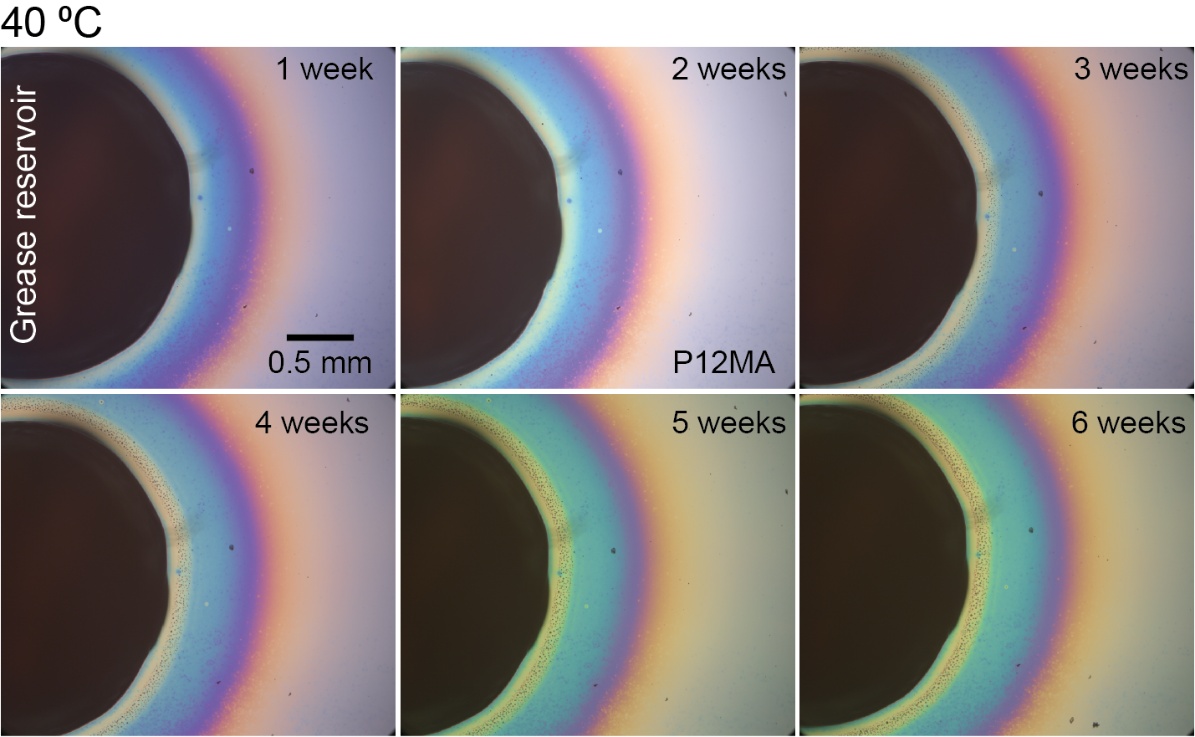


**Fig. S6** Optical top-view images of cylindrical Li/M grease patches (black) deposited on the P12MA brush layer. Samples were heated at 40⁰ C for six weeks; one representative image per week is shown


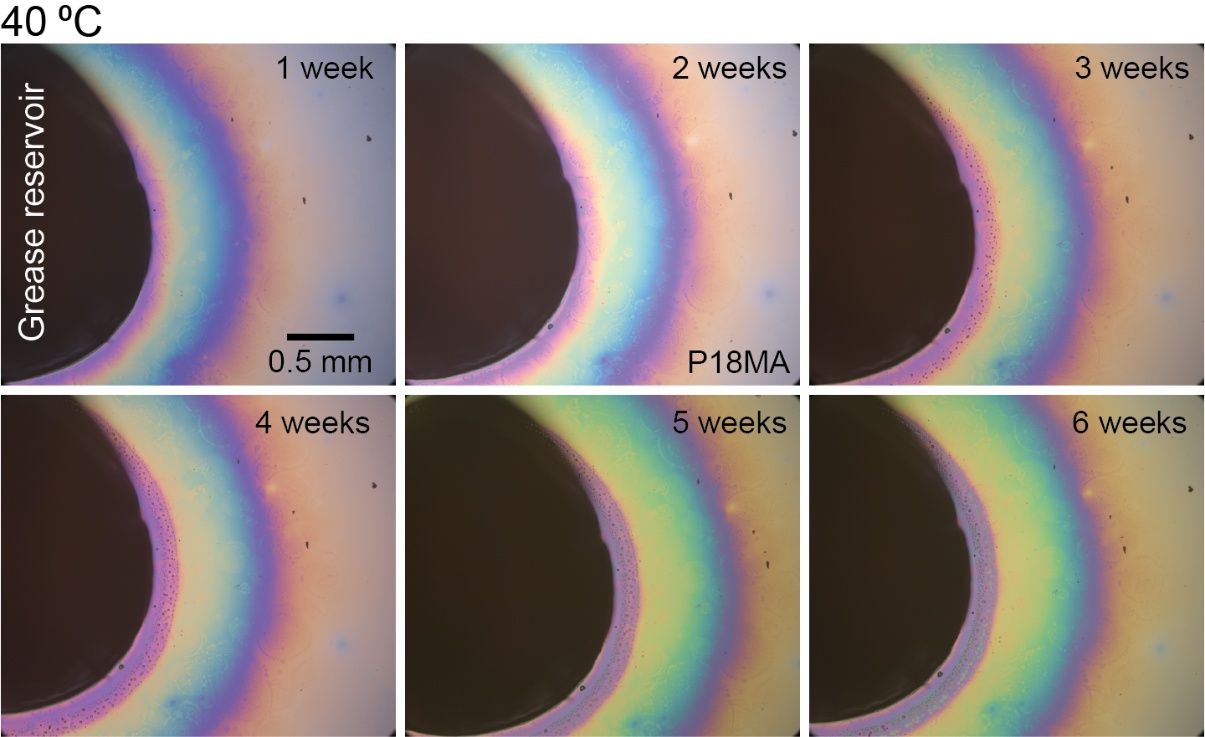


**Fig. S7** Optical top-view images of cylindrical Li/M grease patches (black) deposited on the P18MA brush layer. Samples were heated at 40⁰ C for six weeks; one representative image per week is shown


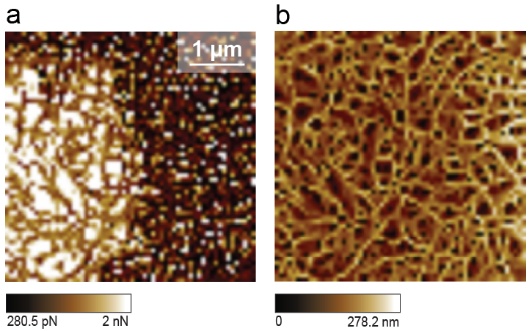


**Fig. S8 a)** Adhesion map obtained from force–volume measurements over the area indicated by the dashed square in Fig. 3b. Dark regions correspond to lower adhesion (dry areas), whereas brighter regions indicate higher adhesion (oil-infused areas). The adhesion map is extracted from the minimum of the retraction curve of each force–distance measurement. **b)** Corresponding surface topography reconstructed from the same force–distance curves. The height map represents the piezo displacement (Δz) at the threshold setpoint force (F_t_) during the approach of the cantilever.
